# The role of ultrasound imaging in vascular compression syndromes

**DOI:** 10.1186/s13089-020-00202-6

**Published:** 2021-02-08

**Authors:** Renato Farina, Pietro Valerio Foti, Andrea Conti, Francesco Aldo Iannace, Isabella Pennisi, Luigi Fanzone, Corrado Inì, Federica Libra, Francesco Vacirca, Giovanni Failla, Davide Baldanza, Stefano Palmucci, Serafino Santonocito, Antonio Basile

**Affiliations:** Radiodiagnostic and Radiotherapy Unit, Department of Medical and Surgical Sciences and Advanced Technologies “GF Ingrassia”, Via Santa Sofia 78, 95123 Catani, Italy

**Keywords:** May–Thurner syndrome, Dunbar syndrome, Nutcracker syndrome, Color Doppler ultrasound, Duplex Doppler ultrasound, Abdominal ultrasound

## Abstract

Vascular compression syndromes are rare alterations that have in common the compression of an arterial and/or venous vessel by contiguous structures and can be congenital or acquired. The best known are the Thoracic Outlet Syndrome, Nutcracker Syndrome, May–Thurner Syndrome, and Dunbar Syndrome. The incidence of these pathologies is certainly underestimated due to the non-specific clinical signs and their frequent asymptomaticity. Being a first-level method, Ultrasound plays a very important role in identifying these alterations, almost always allowing a complete diagnostic classification. If in expert hands, this method can significantly contribute to the reduction of false negatives, especially in the asymptomatic population, where the finding of the aforementioned pathologies often happens randomly following routine checks. In this review, we briefly discuss the best known vascular changes, the corresponding ultrasound anatomy, and typical ultrasound patterns.

## Thoracic Outlet Syndrome (TOS)

### Introduction

**Fig. 1:**
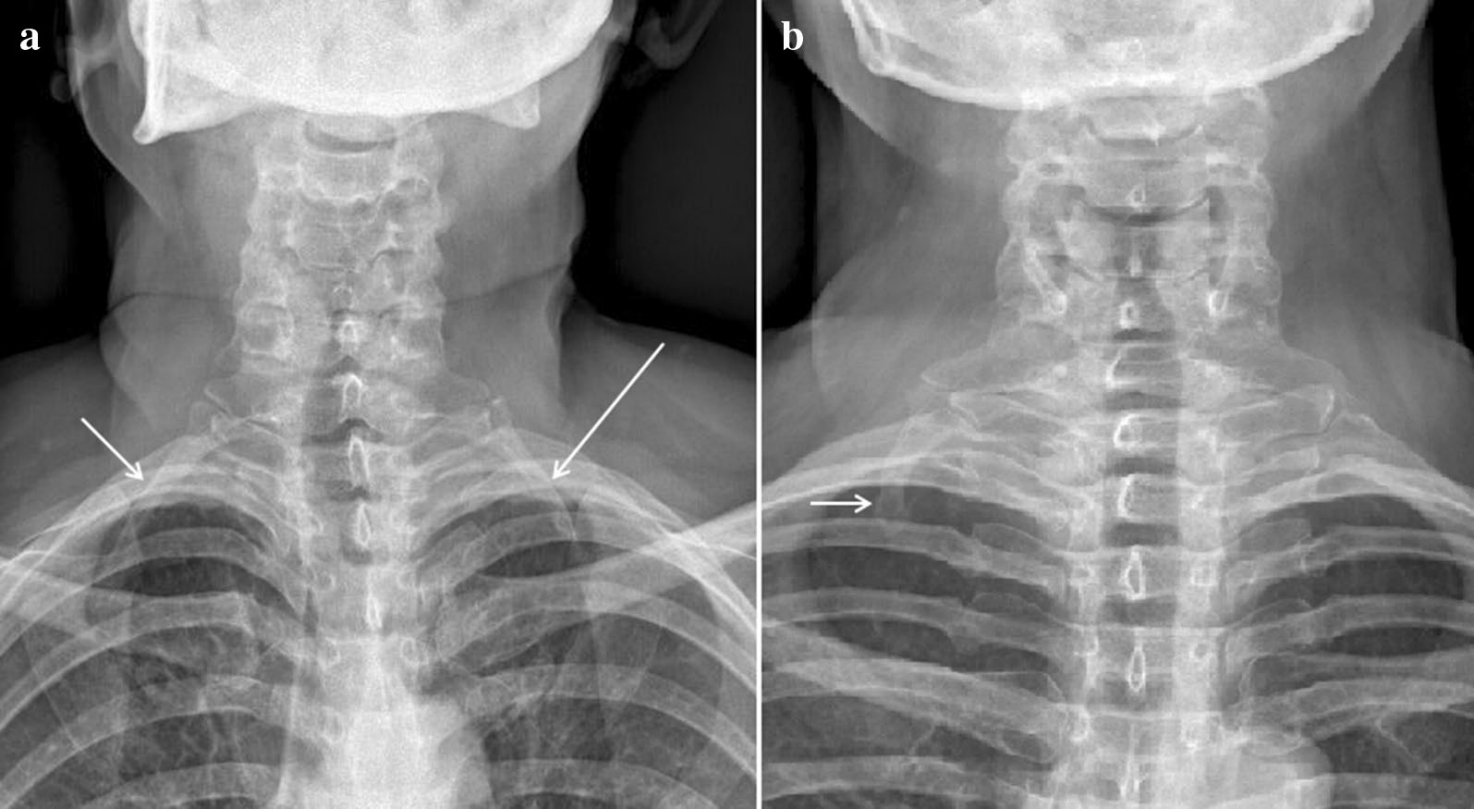
TOS. Standard chest X-ray. Patient with bilateral cervical ribs: **a** right (short arrow) and left (long arrow) cervical rib. Patient with unilateral cervical rib: **b** right cervical rib (arrow)

**Fig. 2:**
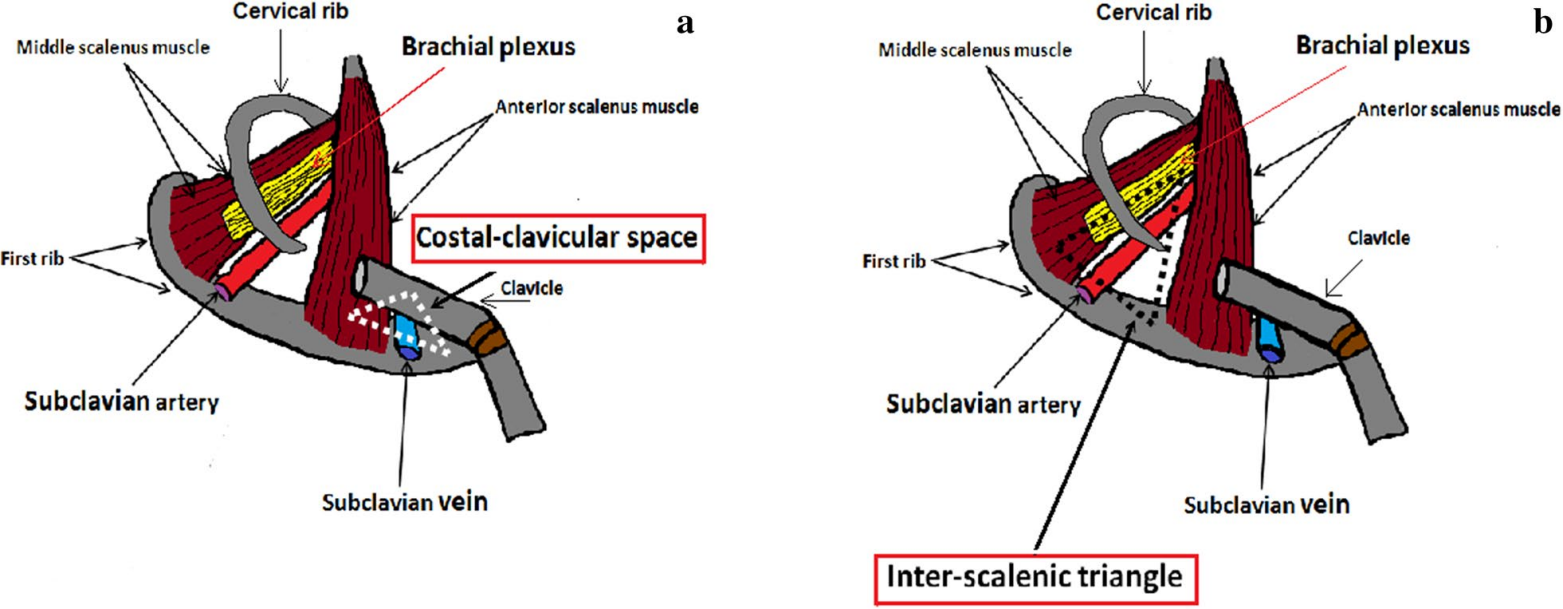
Scheme summarizing the anatomical relationships between the shoulder structures in TOS. **a** Cost-clavicular space delimited inferiorly by first rib, superiorly by clavicle, and anteriorly by anterior scalene muscle. **b** Inter-scalene triangle delimited inferiorly by clavicle, medially by anterior scalene muscle, and laterally by middle scalene muscle

Thoracic Outlet Syndrome (TOS) [1, 2] is a rare pathology of neuro-vascular compression caused by the bilateral (Fig. 1a) or unilateral cervical rib (Fig. 1b) [3] or by hypertrophy of the scalene muscles [4]. The cervical rib is a congenital alteration, often asymptomatic, while hypertrophy of the scalene muscles is generally acquired, frequent in some sports that involve the shoulder muscles. TOS can therefore be bilateral, due to the presence of two cervical ribs and/or to bilateral hypertrophy of the scalene muscles. In most cases, TOS is unilateral. Very rare is the combination of bilateral compression of the artery and subclavian vein, due to the coexistence of two cervical ribs and bilateral hypertrophy of the scalene muscles [5]. The incidence of the disease is higher in females aged between 20 and 50. In TOS, for anatomical reasons, compression of the subclavian vein always takes place at the "cost-clavicular space" [6](Fig. 2a), while compression of the subclavian artery at the level of the "inter-scalene triangle" [7](Fig. 2b). The subclavian artery is almost always compressed by the cervical rib, while the subclavian vein by hypertrophy of the anterior scalene muscle.

### Clinical implications

The cervical rib can compress both the brachial plexus (neurological form) with tingling and/or paresthesia, and the subclavian artery (vascular form) with consequent hypo-perfusion and cyanosis of the upper limb. If compression is caused by hypertrophy of the scalene muscles, it always involves the subclavian vein and causes venous stasis with hypertension, cyanosis, swelling (often in the morning), and pain in the upper limbs. The diagnosis of TOS can be clinical: Adson test [8], Allen test [9], Wright test [10], Halstead maneuver [11], and/or instrumental.

### Instrumental diagnosis

**Fig. 3:**
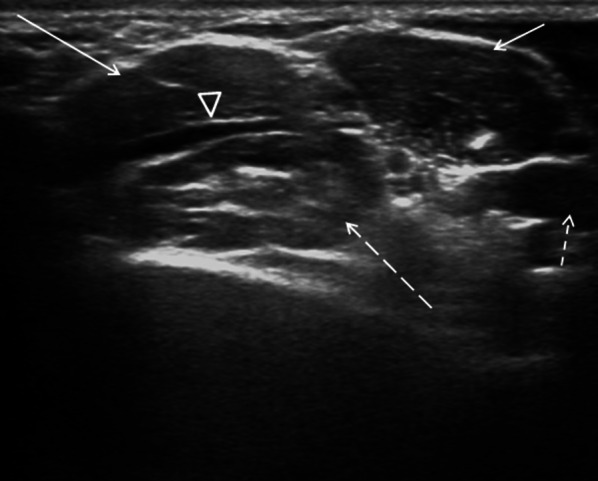
B-Mode US: transverse scan of the cost-clavicular space which highlights the anterior scalene muscle (short arrow), the middle scalene muscle (long arrow), and the posterior scalene muscle (long-dashed arrow). Subclavian artery (head of arrow). Subclavian vein (dashed short arrow)

The imaging is entrusted to Standard Radiography to ascertain the presence of the cervical ribs and to ultrasound for the study of vascular alterations [12]. The Ultrasound is the first-level examination and must be performed with arms raised to 90° and arms lowered (Adson test), to measure the changes in the caliber and flow of the artery and subclavian vein. Generally, by raising the arms to 90°, the arterial and/or venous compression appears or accentuates and with them the symptomatology. The Ultrasound examination must be performed using both B-Mode Ultrasound (US) for the scalene muscles and cervical ribs morphological study (Fig. 3); Color Doppler US and Duplex Doppler US for the flowmeter study [13].

Color Doppler US of the subclavian vein must be performed at the level of the "costal-clavicular space", where compression occurs, which is delimited below by first rib, above by clavicle and anteriorly by anterior scalene muscle. The subclavian artery study must instead be carried out at the level of the "inter-scalene triangle" which is delimited inferiorly by clavicle, medially by anterior scalene muscle and laterally by middle scalene muscle. During the Adson test, the caliber and the flow of the vessels must be measured.

**Fig. 4:**
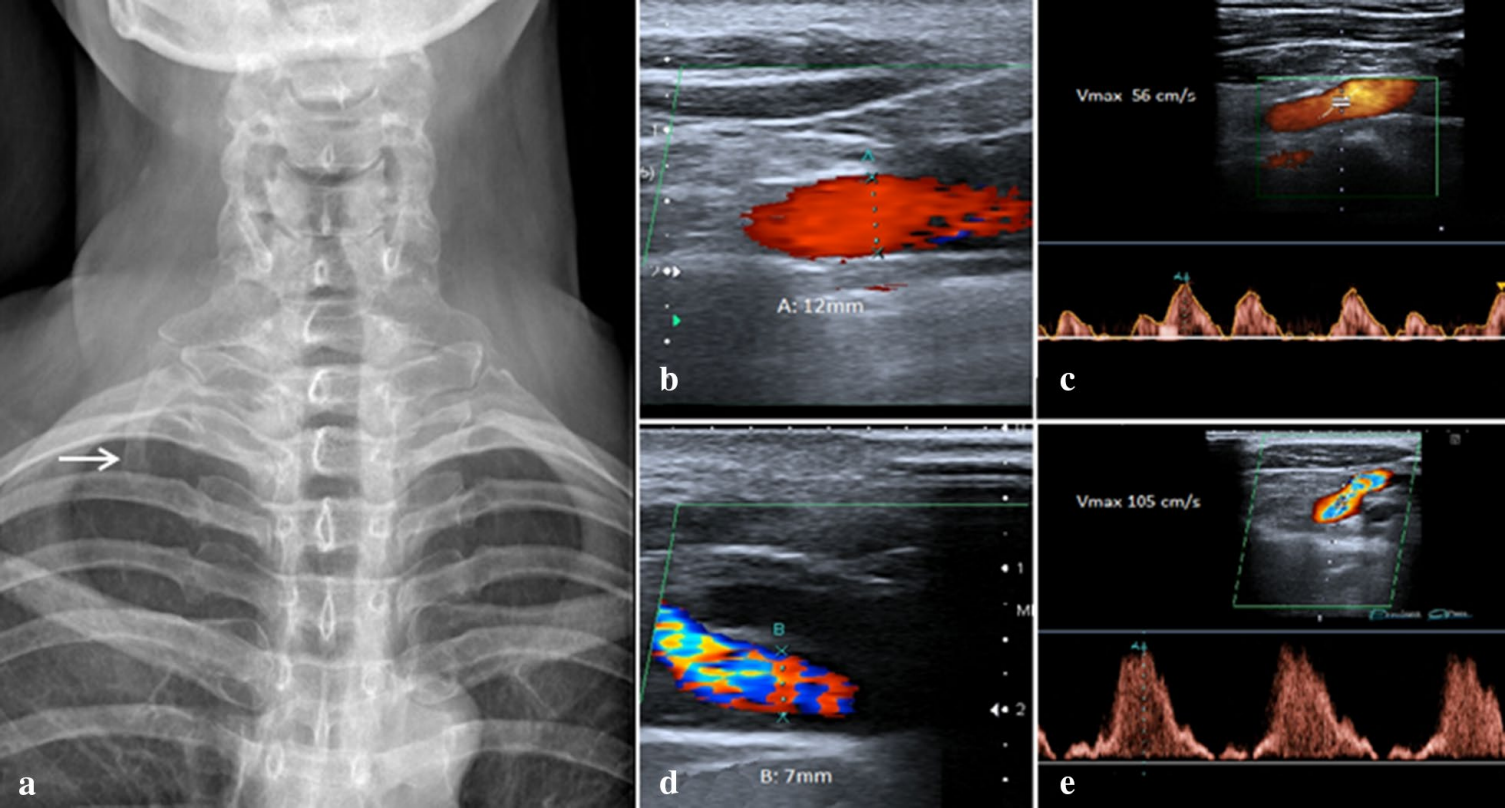
**a** Standard radiography showing a cervical rib on the right (arrow). **b** Color Doppler US examination, performed with lowered arms, shows a regular diameter (12 mm) and a regular flow-C of the right subclavian artery. **d** Color Doppler US examination with raised arms shows artifacts due to turbulent flux. E: Duplex Doppler US shows increase in peak speed (105 cm/s)

**Fig. 5:**
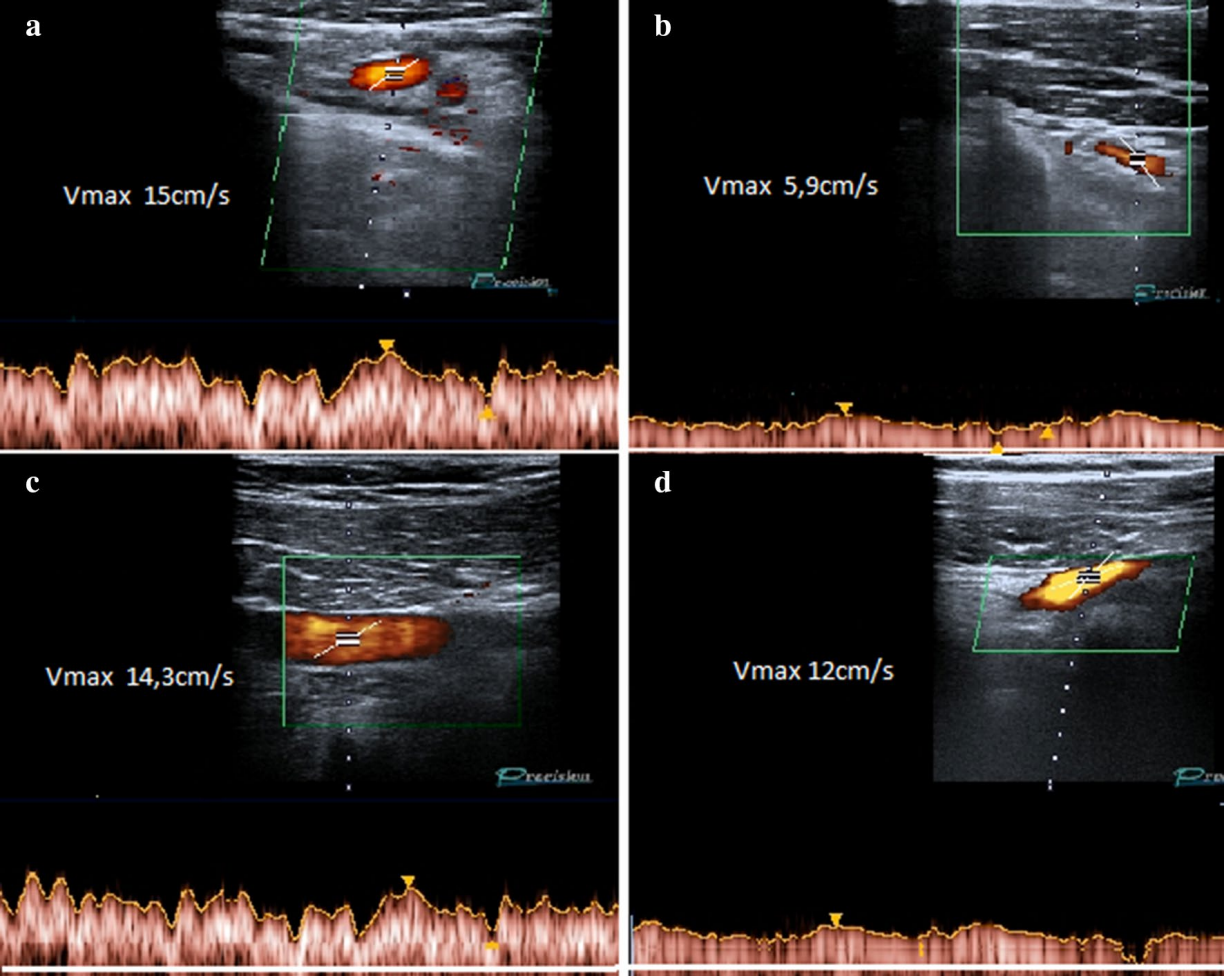
Hypertrophy of the right anterior scalene muscle. **a** Duplex Doppler US examination of the right subclavian veins, with lowered arms, shows a regular diameter and flow. **b** With arms raised to 90° Duplex Doppler US shows a peak speed reduction due to compression by the anterior scalene muscle. **c** Duplex Doppler US of left subclavian vein, with lowered arms, shows a regular caliber and flow. **d** Which remain regular even with arms raised to 90°

In subclavian vein compression upstream of the stenosis, a slowing of the peak flow with consequent venous hypertension is observed (Fig. 4a–e). When the compression involves the subclavian artery, it is possible to observe a progressive reduction in the caliber of the vessel passing from the position with lowered arms to that with raised arms and an increase in the peak speed proportional to the degree of stenosis; if the stenosis is severe, very high speeds and aliasing artifacts are observed with Color Doppler US and Duplex Doppler US, due to the turbulent flow in the stenotic tract (Fig. 5a–d) Magnetic Resonance Imaging (MRI) can highlight the main signs of TOS, but is mainly used in children to avoid the radiological risk related to the ionizing radiation [14]. Multidetector Computed Tomography (MDCT) is used in the diagnosis of TOS for its overview and high accuracy for vascular structures [15]; moreover, even if burdened by radiological risk, recent technological developments have made it possible to lower radiation doses, without compromising image quality [16].

### Treatment

Patient treatment can be surgical with cervical rib resection [17] and scalenectomy [18], or conservative with physiotherapy, orthotics, and taping [19]. The above treatments are all aimed at reducing arterial and/or venous vascular compression.

## Nutcracker Syndrome (NCS)

### Introduction

**Fig. 6:**
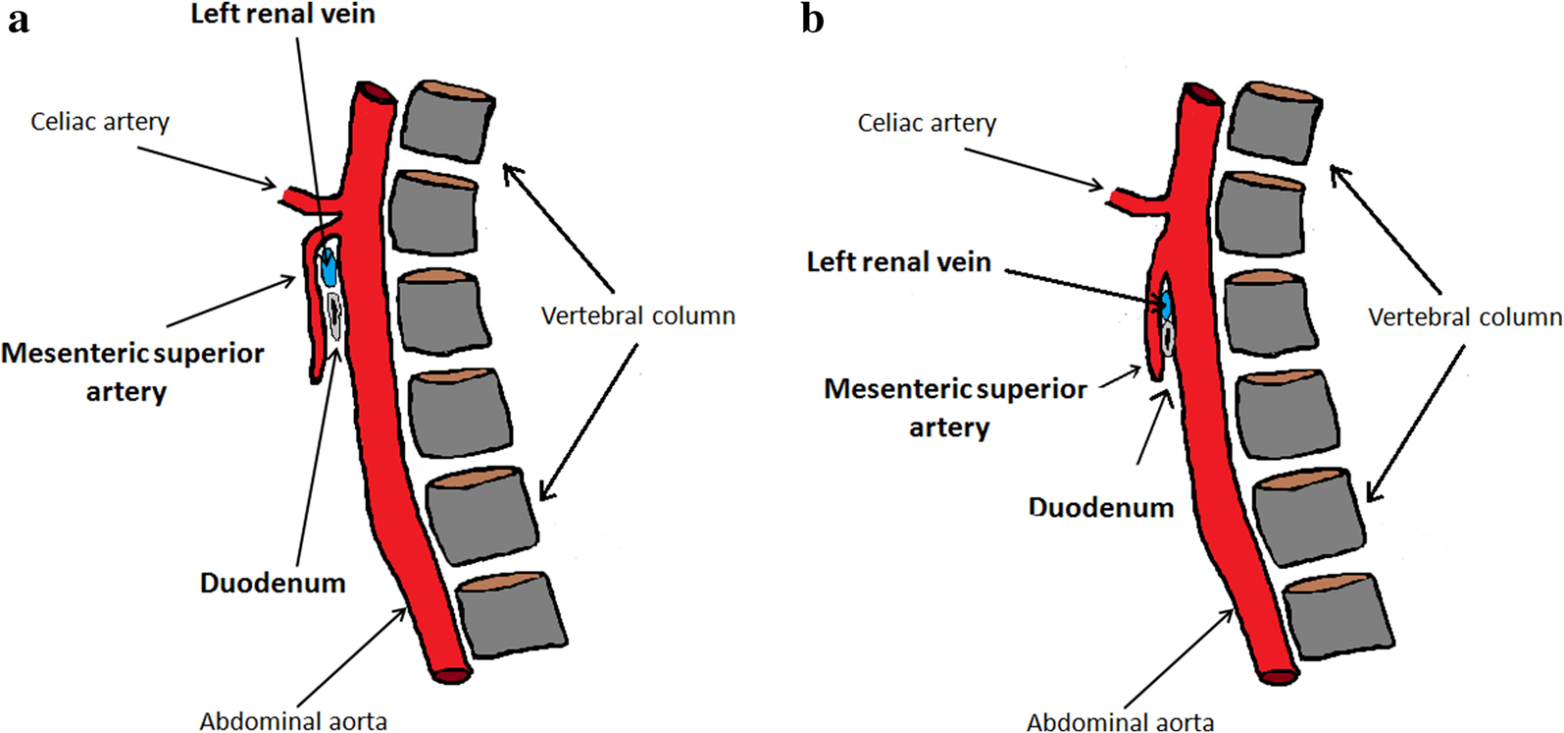
NCS. Scheme summarizing that describes the relationships between the aorto-mesenteric angle, left renal vein, and duodenum. **a** Healthy patient with regular aorto-mesenteric angle. **b** Aorto-mesenteric angle lower than 22° which causes compression of the left renal vein

NCS, also known as left renal vein entrapment syndrome, is a rare vascular alteration due to compression of the left renal vein in the transition between the abdominal aorta and the superior mesenteric artery [20]. It was first described by Wilkie [21]. This disease is caused by the reduction in the angle between the abdominal aorta and the superior mesenteric artery that originates at an angle of less than 22 degrees, maintaining a distance to the aorta of less than 8 mm. The reduced angle involves the structures that pass through this anatomical space, namely the duodenum and the left renal vein which undergo compression proportionate to the reduction of the aorto-mesenteric angle (Fig. 6a, b). Isolated stenosis of the left renal vein is commonly called "NCS ", while isolated compression of the duodenum "Wilkie Syndrome" (WS). The two alterations can combine or occur in isolation. In most cases, the compression of the renal vein arises anteriorly to the aorta, while in much rarer cases, it occurs posteriorly and happens when the renal vein is retro-aortic; in this case, compression occurs between the spine and the abdominal aorta [22]. The incidence of the disease is probably underestimated considering that compression is often asymptomatic and that there are cases of unknown proteinuria and hematuria that could be caused by NCS. NCS can affect all age groups, but it prevails in very thin young people [23]. The Syndrome can be congenital or acquired. In the acquired form, the greatest prevalence is in anorexic patients and is due to the reduction of the peri-vascular adipose tissue which results in a narrowing of the aorto-mesenteric angle; in these patients, vomiting, which is initially self-induced, following the onset of duodenal compression (WS), becomes organic and contributes to the progressive worsening of clinical conditions [24].

### Clinical implications

In NCS not combined with WS, clinically patients may have different clinical manifestations, ranging from asymptomatic hematuria to proteinuria, nephrovascular hypertension, left flank pain, and secondary varicocele [25]. The most commonly reported symptom is hematuria due to rupture of thin-walled varices due to venous hypertension [26]. If compression involves the duodenum, vomiting, sub-occlusive crisis, and weight loss may occur and the most constant symptom is post-prandial vomiting. The combination of the two syndromes can manifest with all the above symptoms.

### Instrumental diagnosis

**Fig. 7:**
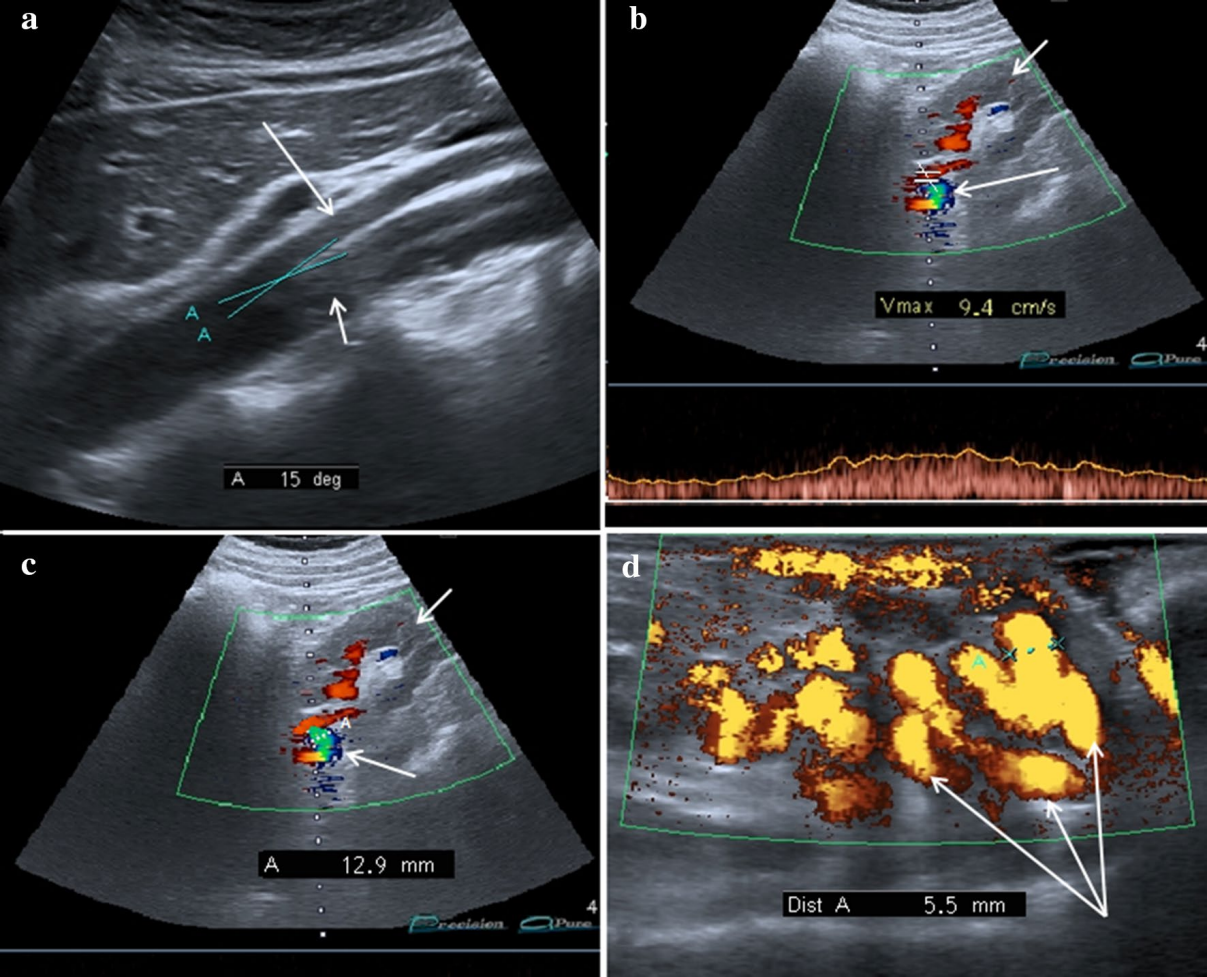
B-Mode US: longitudinal sub-xiphoid scan of the abdominal aorta performed in supine decubitus. **a** Measurement of the aorto-mesenteric angle "A" in patient with NCS. Abdominal aorta (short arrow). Superior mesenteric artery (long arrow). **b** Duplex Doppler US shows a peak speed reduction in left renal vein. **c** Measurement of the left renal vein diameter. **d** Power Doppler US shows varicosity of the gonadal plexus (vein diameter 5.5 mm)

**Fig. 8:**
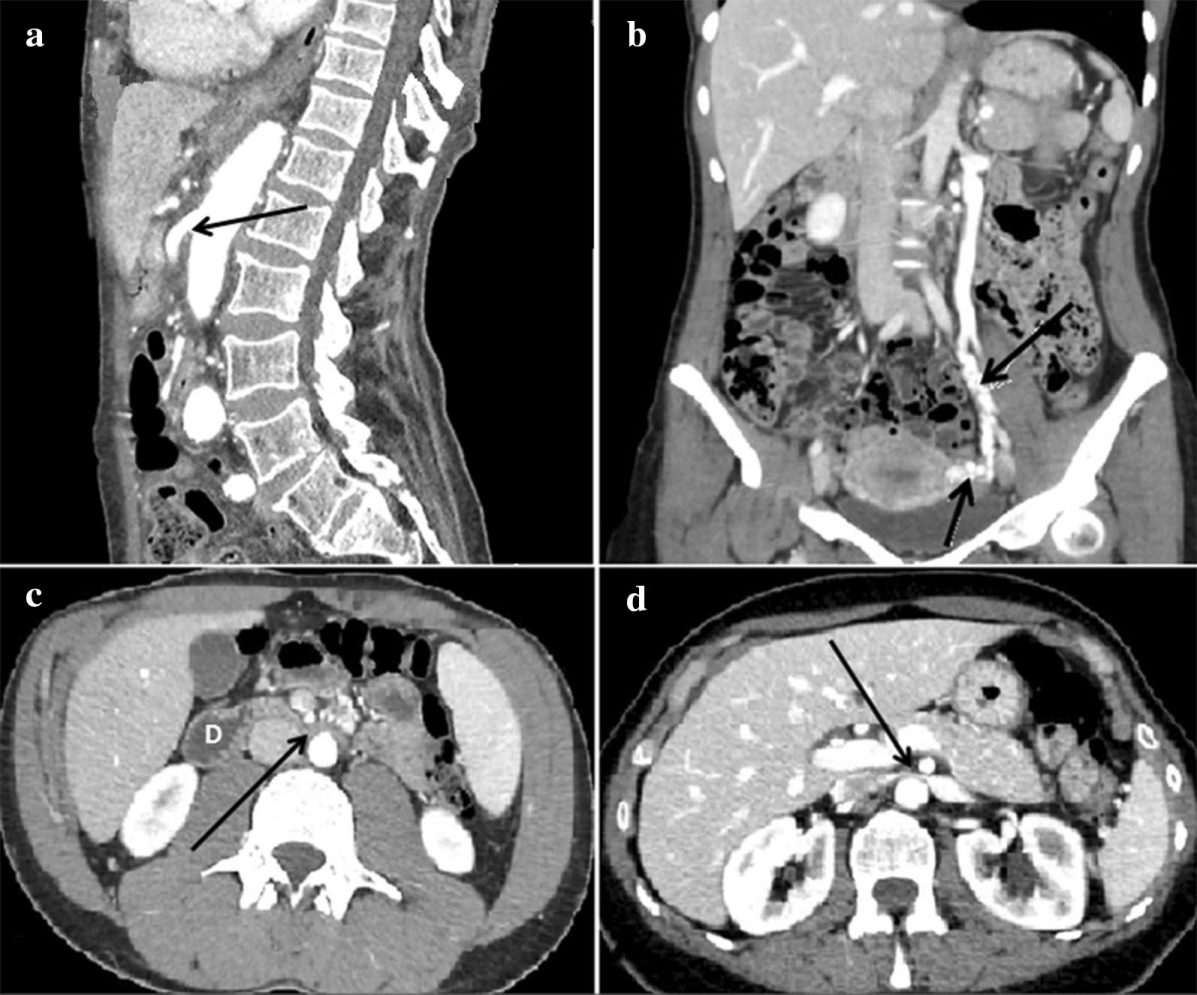
Abdomen MDCT examination. **a** The reconstruction according to a sagittal plane shows the characteristic pattern with aorta and superior mesenteric artery "beak-like" appearance (black arrow). **b** The coronal plane reconstruction shows dilation of the gonadal vein and gonadal plexus (arrow). **c** The axial plane reconstruction shows a stenosis of duodenum (arrow). "D": Duodenum. **d** The axial plane reconstruction shows a stenosis of the left renal vein (arrow)

**Fig. 9:**
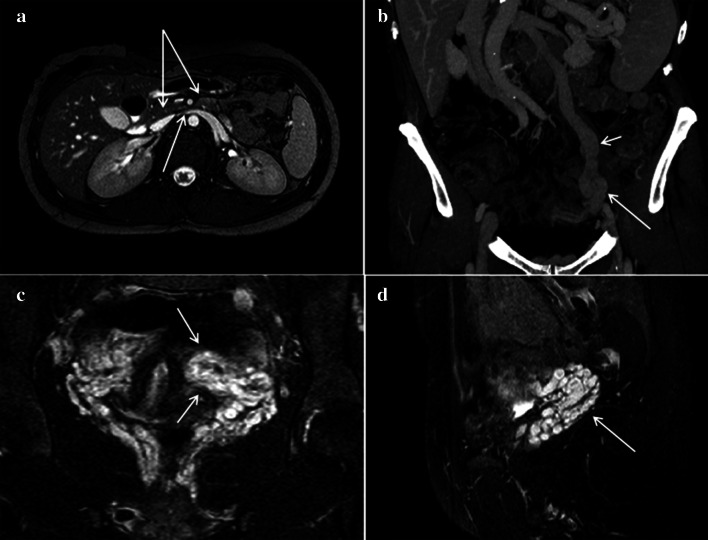
Abdomen MRI examination. **a** The axial plane reconstruction shows a stenosis of the left renal vein and duodenum (arrows) in the aorto-mesenteric angle (arrow). **b** The coronal plane reconstruction shows gonadal vein (short arrow) and gonadal plexus (long arrow) dilatation. **c** The coronal plane reconstruction shows the varicosities of the gonadal plexus (arrow), also evident in the reconstruction according to a sagittal plane (arrow)—D

Ultrasound is the first-level examination for the diagnosis of NCS, it allows you to accurately measure the aorto-mesenteric angle and the aorto-mesenteric distance (Fig. 7a) (Clip 1. NCS. B-Mode US of the AO) [27]; it can also measure the flow (Fig. 7b) and the caliber of pre-stenotic tract of the left renal vein (Fig. 7c). Pelvic Ultrasound examination can highlight varicosities of the pampiniform and/or gonadal plexus (Fig. 7d) due to stasis and hypertension of the left renal vein. Ultrasound therefore allows a complete diagnostic framework of the NCS but not of the WS, for the diagnosis of which integration with other imaging methods such as MR-Enterography [28], Fluoroscopy [29], and Ecoendoscopy [30] is necessary. MDCT can demonstrate compression and pre-stenotic dilation of the left renal vein, as well as the presence of varicocele. An advantage of MDCT is the possibility of highlighting also the stenosis of the duodenum and the intestinal dilation upstream of the stenosis. A pathognomonic sign of NCS in MDCT is the "Beak sign" that is the origin of the superior mesenteric artery from the aorta with an acute angle also known as "hooked appearance" evident in the sagittal reconstructions (Fig. 8a–d) [31]. MRI can highlight all pathognomonic signs of NCS (Fig. 9a–d); compared to MDCT, it is not burdened by radiological risk, but is less sensitive for the evaluation of duodenal stenosis [32, 33].

### Treatment

**Fig. 10:**
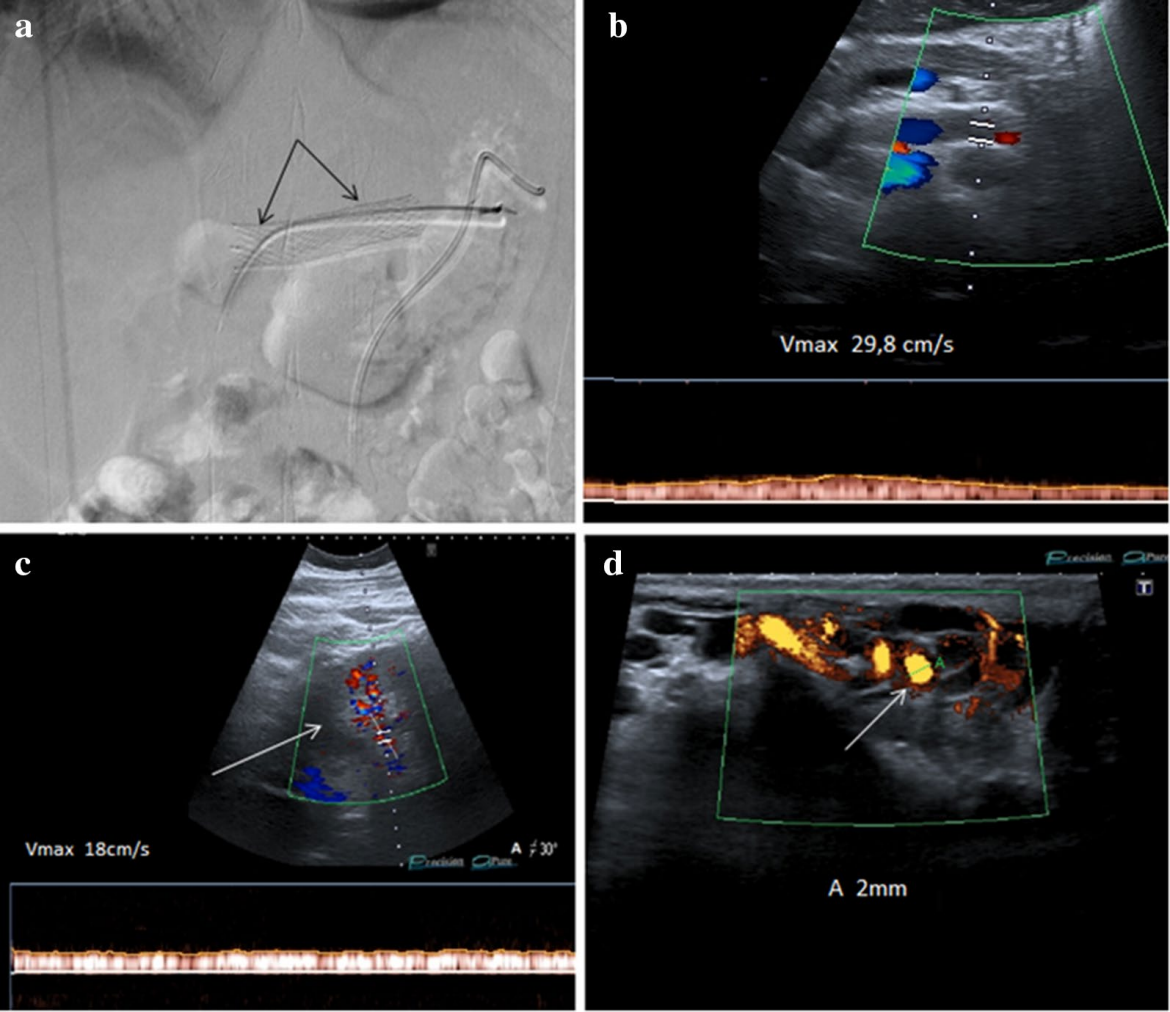
**a** This angiographic image shows the endovascular stent after positioning into the left renal vein (arrows). **b** Duplex Doppler US highlights the patency of the vascular stent showing a flow with a peak velocity of about 29.8 cm/s. **c** After stenting, Color Doppler US show a increased flow of the left renal vein (18 cm/s). **d** Power Doppler US shows a regular caliber of the pampiniform plexus vein (diameters of 2 mm)

The choice of treatment should be based on the clinical presentation, physical condition, and severity of left renal vein stenosis. Conservative treatment, of choice, when possible, consists in restoring the normal layer of peri-vascular fat tissue with a high calorie diet [34]. The other two therapeutic approaches are surgical treatment [35, 36] and endovascular stenting treatment [37]. The surgical treatment consists in overcoming the stenosis with the resection of the first jejunal loop and the retrovascular duodenum followed by the anastomosis between the duodenum and the second jejunal loop which are anteriorized. In recent years, however, the use of interventional procedures with stenting of the left renal vein [38] has led to a significant reduction in surgical treatments, much more invasive and with greater complications. The positioning of the endovascular stent in the left renal vein causes the restoration of the normal aorto-mesenteric angle with resolution of the venous compression and all the alterations related to it (Fig. 10a–d). Power Doppler US (Clip 2. NCS. After stenting, power Doppler US that shows flow inside the stent), Duplex Doppler US (Clip 3. NCS. After stenting, duplex Doppler US that shows flow inside the stent), and selective Angiography (Clip 4. NCS. Selective Angiography demonstrates stent patency) can be used to check the patency of the endovascular stent. The absence of treatment can predispose to left renal venous thrombosis with consequent renal damage up to the loss of the organ.

## May–Thurner Syndrome (MTS)

### Introduction

**Fig. 11:**
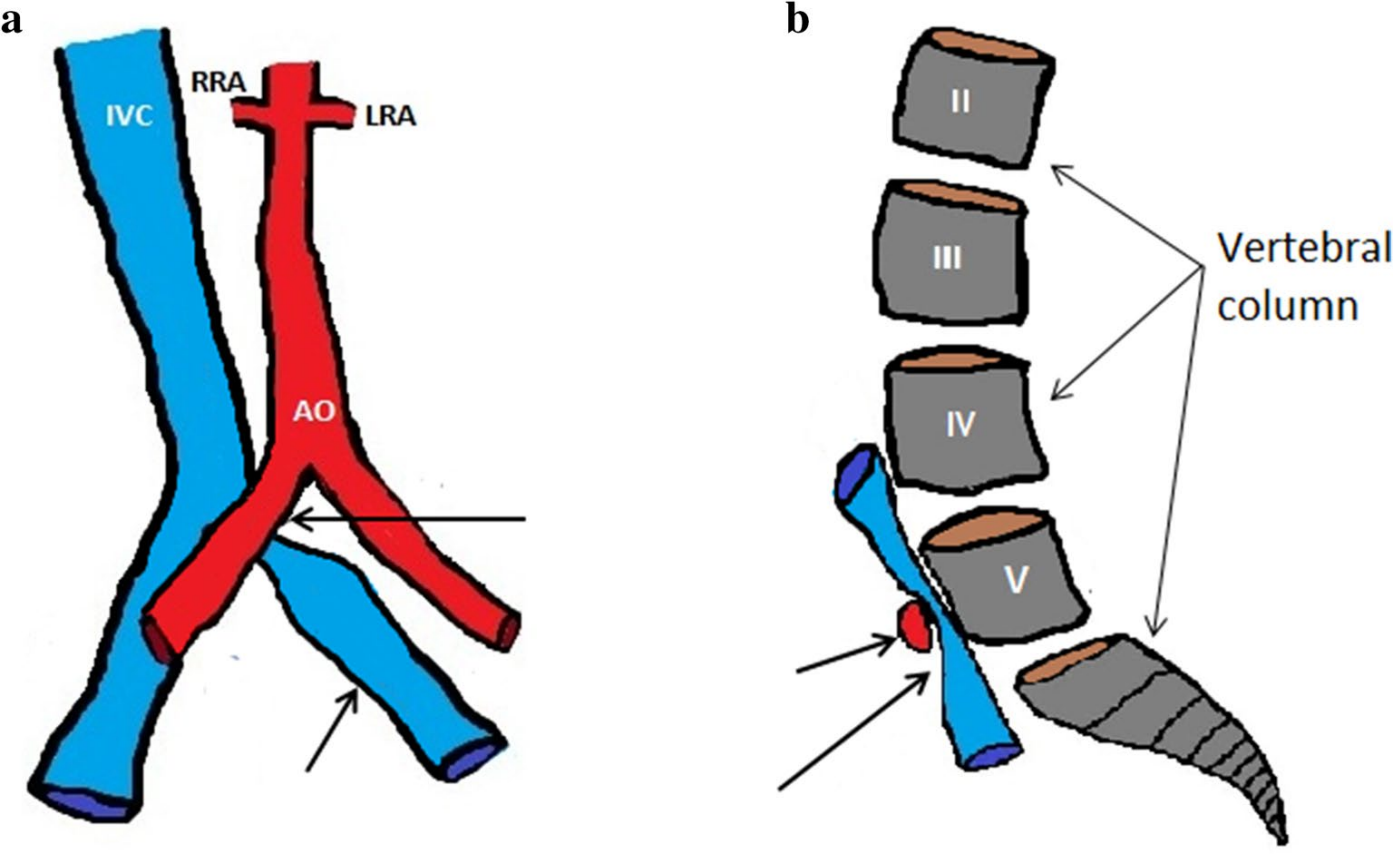
MTS. Scheme summarizing describing the main anatomical structures involved in the syndrome. **a** Left common iliac vein compression (short arrow) by the right common iliac artery (long arrow). *AO* Abdominal aorta. IV **c** Inferior cava vein. RR **a** Right renal artery. *LRA* Left renal artery. **b** This illustration shows the right common short iliac artery which compresses the left common iliac vein (long arrow) against the spinal column

MTS [39] also known as Cockett Syndrome [40] is caused by chronic compression of the left common iliac vein against the lumbar spine by the right common iliac artery (Fig. 11a, b). Compression of the left common iliac vein can generate various degrees of venous hypertension and can predispose the left lower limb to thrombosis. The exact incidence of the disease is unknown both, because it can be asymptomatic [41] and due to the specificity of the symptoms. In 1851, Virchow noted a five times higher incidence of deep vein thrombosis on the left side than deep vein thrombosis on the right side. The anatomical variant responsible for this discovery was described in 1908 by McMurrich; however, it was May and Thurner in 1957 to better frame the mechanisms of the Syndrome, describing the formation of "spurs" in the left common iliac vein as a consequence of chronic compression at work of the right common iliac artery against the spine. The combination of arterial pulsations and mechanical compression by the right common iliac artery would cause hypertrophy of the intimate, with consequent accumulation of elastin and collagen that form the so-called "spurs" responsible for the narrowing of the vascular lumen. In most cases (84%), the right common iliac artery compresses the left common iliac vein, but compression of the right common iliac vein by the ipsilateral common iliac artery has also been described [42]. Compression generally occurs against the fifth lumbar vertebra, but also against the fourth lumbar vertebra has been described [43]. Other causes of compression of the left common iliac vein caused by the bladder [44], endometriosis [45], a penile prosthesis reservoir [46], and aneurysm of the common iliac artery [47, 48] have been described in the literature.

### Clinical implications

Symptomatology in MTS is related to the degree of stenosis of the left common iliac vein and the presence or absence of deep vein thrombosis. In the milder degrees of compression, it can be asymptomatic, while in the most severe degrees, patients can experience: swelling of the left lower limb, pain, venous claudication, deep vein thrombosis, and up to the most serious complication which is pulmonary embolism.

### Instrumental diagnosis

**Fig. 12:**
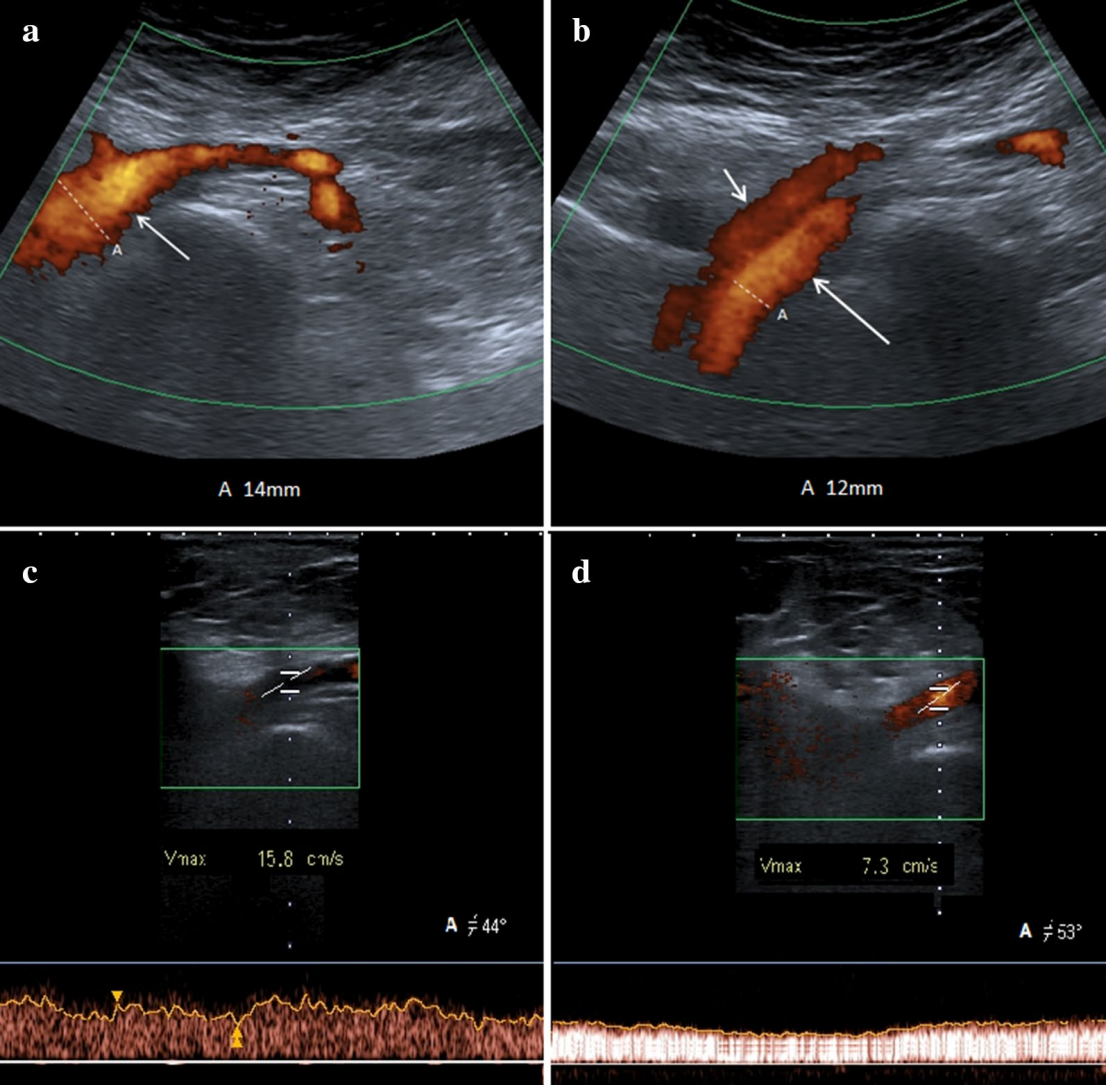
Power Doppler US and Duplex Doppler US of common iliac veins. **a** Left common iliac vein (arrow) dilatation in the pre-stenotic tract (14 mm). **b** Right common iliac vein (long arrow) with regular diameter (12 mm). Right common iliac artery (short arrow). **c** Duplex Doppler US shows a regular peak speed in the post-stenotic tract of the left common iliac vein (15.8 cm/s) and peak speed reduction in the pre-stenotic tract (7.3 cm/s) -D

**Fig. 13:**
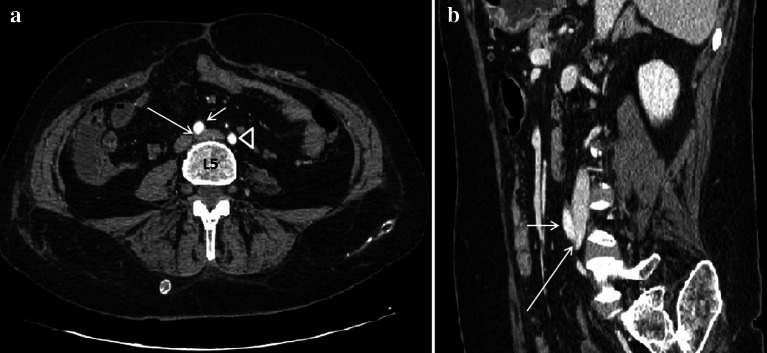
Abdomen MDCT examination. **a** The axial plane reconstruction shows a stenosis of the left common iliac vein (long arrow) by the right common iliac artery (short arrow) against the vertebral column. Fifth lumbar vertebra (L5). Left common iliac artery (head arrow). **b** The sagittal plane reconstruction shows the point where the right common iliac artery (short arrow) compresses the left common iliac vein (long arrow) against the vertebral column

**Fig. 14:**
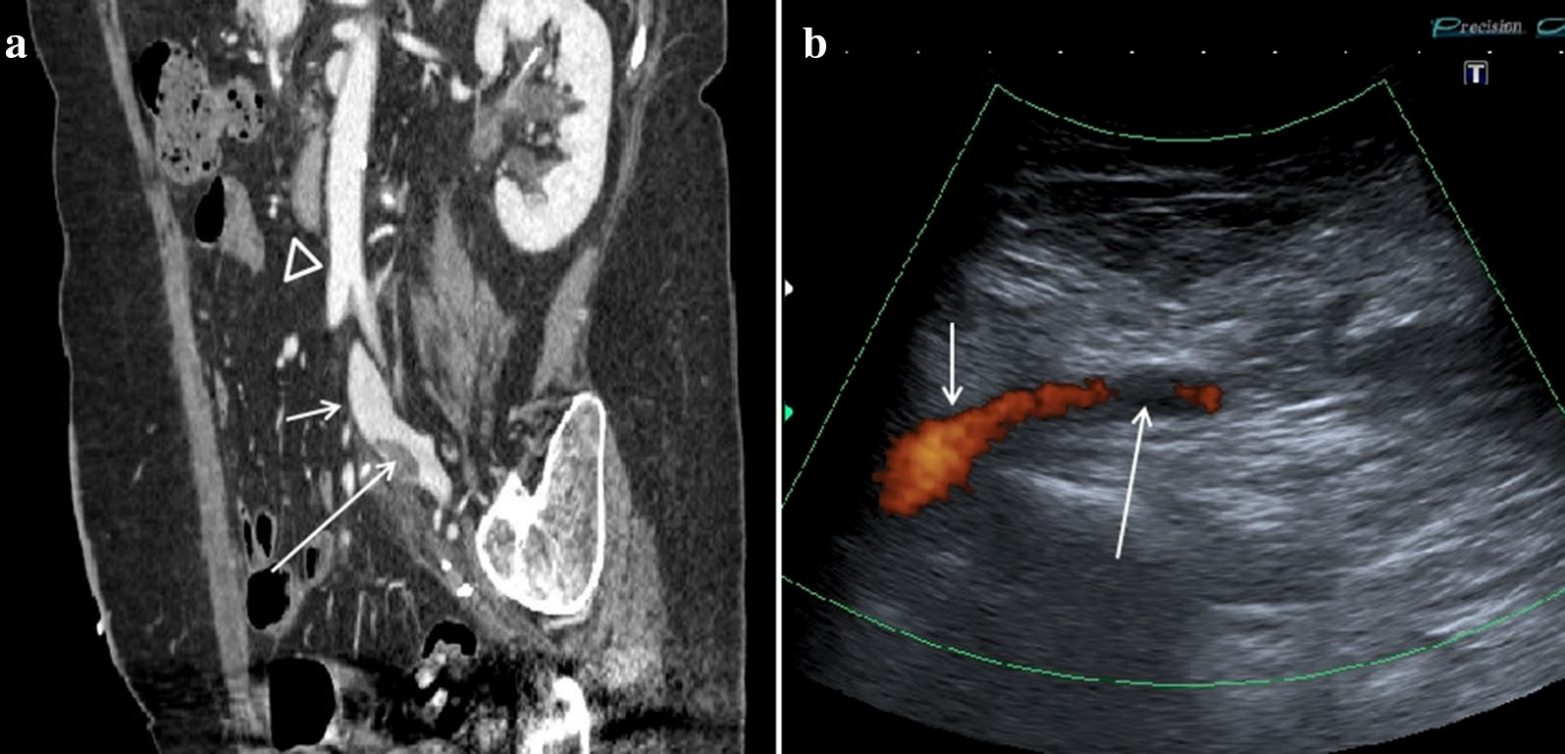
Abdominal MDCT examination. **a** The sagittal plane reconstruction shows an enhancement defect (long arrow) in the left common iliac vein (short arrow) due to a thrombus. Abdominal aorta (head arrow). **b** Power Doppler US shows a thrombus (long arrow) in the left common iliac vein (short arrow)

Ultrasound represents the first-level imaging method thanks to the high sensitivity, low costs, equipment availability, and absence of risks. Color Doppler US, Power Doppler US, and Duplex Doppler US can highlight deep venous thrombosis and measure their extension. Unlike other imaging methods, Ultrasound allows you to measure the left common iliac vein flow by providing an estimate of stenosis severity and venous hypertension degree: the ratio between downstream flow and upstream flow of the stenosis can in fact give an indirect measure of the stenosis degree [49] (Fig. 12a–d). Lower limb MDCT can demonstrate compression of the left common iliac vein by the right common iliac artery (Fig. 13a, b) (Clip 5. MTS. Power Doppler US showing stenosis of the left common iliac vein) and allows to exclude other causes of compression, and it can also highlight the presence of venous thrombosis (Fig. 14a, b).

Intravenous ultrasound venography is the most accurate way to define the extension and type of morphological lesions of the iliac vein [50]. MRI, like MDCT, can demonstrate compression of the left common iliac vein by the right common iliac artery and rule out other causes of compression.

### Treatment

Endovascular stenting [51–53] has progressively replaced surgical thrombectomy, because it is less invasive and also represents the best therapeutic approach when pharmacological thrombolysis has contraindications. Short-term or long-term thrombolytic, anticoagulant prophylaxis, and vascular stenting currently seem to represent the treatment of choice for symptomatic MTS and hemodynamically significant stenosis of the left common iliac vein. According to the authors, in patients with thrombosis and edema of the lower limb, endovascular treatment is successful in 91% of patients. In patients with acute thrombosis, however, direct trans-catheter thrombolysis is still performed [54]. Other types of intervention have recently been reported, such as "radiofrequency thermocoagulation" [55] not yet supported, however, by sufficient case studies.

## Dunbar Syndrome (DS)

### Introduction

**Fig. 15:**
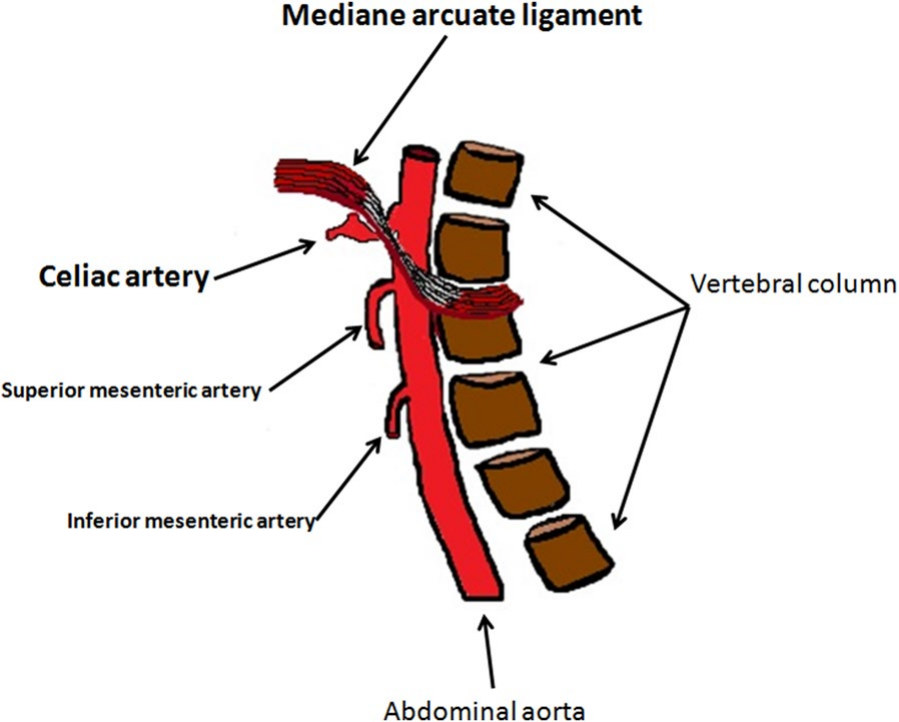
DS. Scheme summarizing of the anatomical structures involved in DS. More caudal course of the MAL that compresses the CA in the expiratory apnea phase

DS [56], also known as median arcuate ligament syndrome (MALS) [57], is a vascular alteration caused by compression of the celiac artery (CA) and/or surrounding neural ganglion by the median arcuate ligament (MAL) of the diaphragm. In healthy patients, the MAL runs cranially to the ostium of the CA; in some patients, however, it runs more caudally, always above the origin of the CA, causing stenosis (Fig. 15). The cause of this alteration is still unknown. There are congenital factors in the literature [58], but cases in which it occurred following surgery [59] are also reported. The syndrome prevails in women between the ages of 30 and 50 [60]. The incidence is estimated at around 2 for every 100,000 patients.

### Clinical implications

This vascular alteration is very difficult to diagnose, since the clinical manifestations depend on the degree of stenosis and are often non-specific; moreover, this disease is not well known by operators. Compression of the CA, if mild, can be asymptomatic and can go unnoticed in simple routine checks. When stenosis is significant, the resulting chronic ischemia becomes symptomatic and can change with the respiratory acts; in fact, it generally increases with forced exhalation which causes a relaxation in the diaphragm and a lowering of the MAL. In more severe cases, ischemia no longer changes with respiratory acts. Symptomatology can include non-specific symptoms such as diarrhea, back-sternal pain, vomiting, swelling, and nausea, but there is a typical clinical presentation represented by a triad: weight loss, post-prandial abdominal pain (94.4%), and epigastric murmur [61, 62]. The first two symptoms are more frequent and linked to each other, because the transient functional ischemia that occurs during digestion causes pain and induces patients to limit meals causing weight loss.

### Instrumental diagnosis

The diagnosis must be based on imaging and clinic, and must exclude pathologies that have a similar clinical presentation, such as cholecystitis, pancreatitis, neoplasms of the digestive tract, peptic ulcer, gastritis, appendicitis, hepatitis, intestinal ischemia, etc. To be considered DS, it must be symptomatic; therefore, in asymptomatic patients, there is no mention of DS but only of a vascular alteration well compensated by collateral circulation. Color Doppler US and Duplex Doppler US are considered to be first-level tests for diagnosis [63]. Second-level exams are represented by MDCT [64], MRI [65], and selective Angiography [66].

**Fig. 16:**
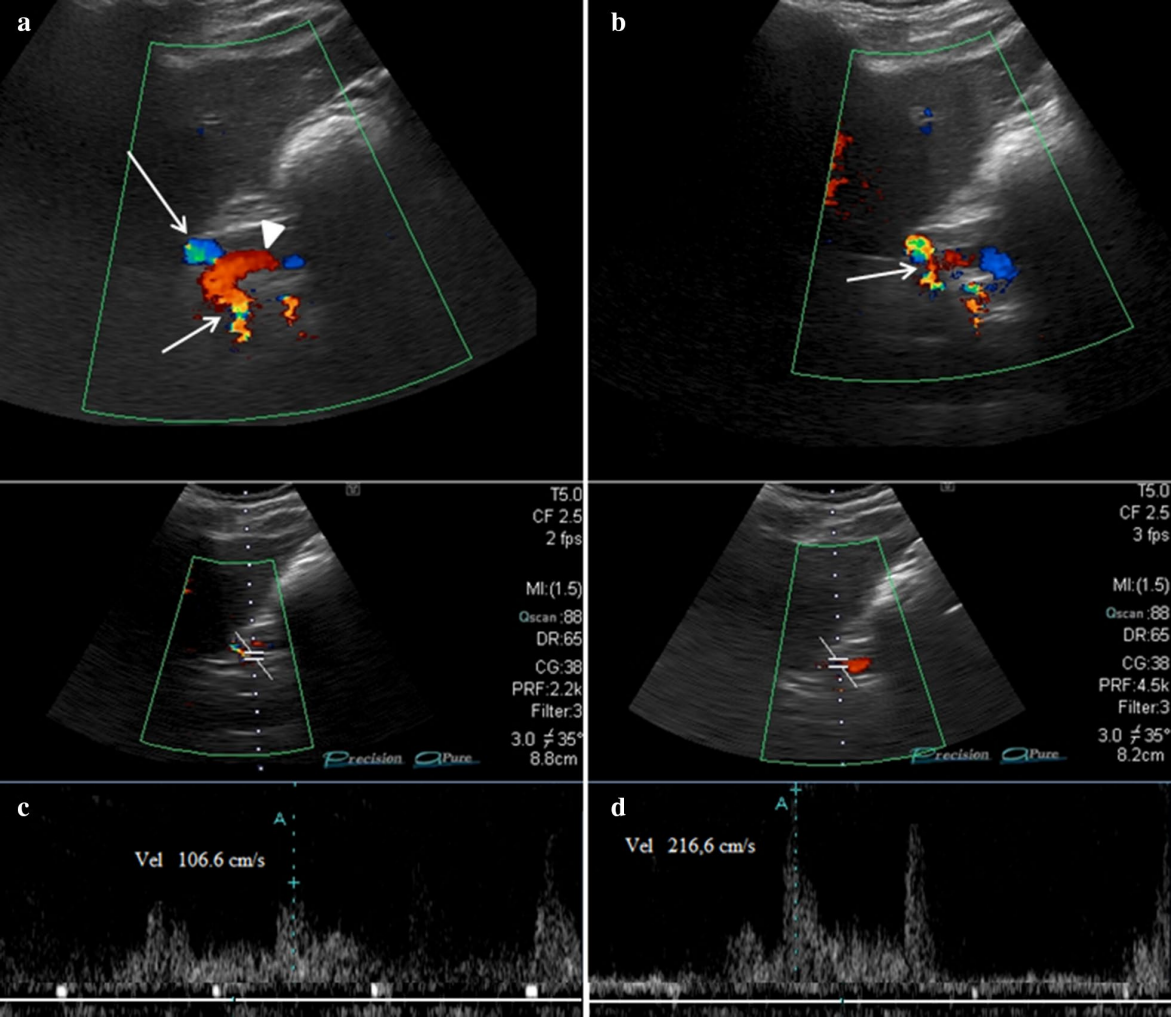
Abdominal MDCT examination. **a** The axial plane reconstruction shows a stenosis of CA (arrow), origin off the abdominal aorta. **b** The sagittal plane reconstruction shows stenosis of CA with the "Hooked appearance" (long arrow). AO: Abdominal aorta. Superior mesenteric artery (short arrow)

**Fig. 17:**
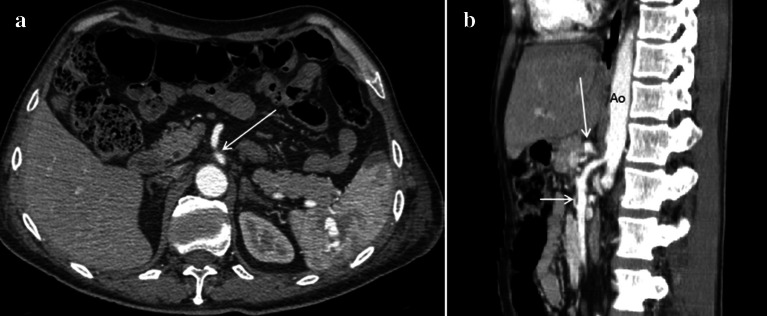
Abdomen MRI examination. **a** The axial plane reconstruction shows a stenosis of CA (long arrow); origin off the abdominal aorta (short arrow). **b** The axial plane reconstruction, cranially to the CA origin, shows the MAL (arrows). Abdominal aorta (short arrow)

**Fig. 18:**
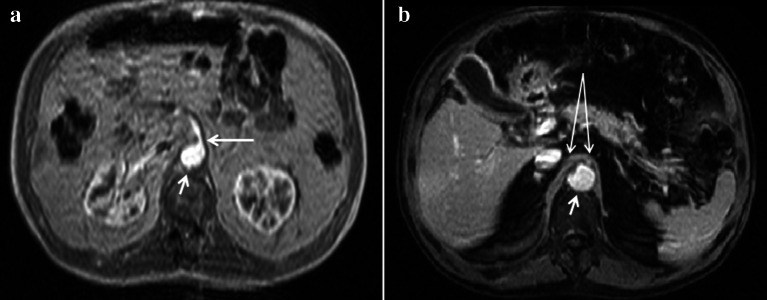
Transverse sub-xiphoid Ultrasonographic scan. **a** Color Doppler US performed in inspiratory apnea that shows a regular diameter of the CA (short arrow). Epatic artery (long arrow). Splenic artery (head of arrow). **b** Color Doppler US performed in expiratory apnea that shows severe stenosis at the origin of the CA with aliasing due to turbulent flow and high-speed peak. **c** Duplex Doppler US of the CA performed in inspiratory apnea that shows a slight increase in peak speed. **d** Duplex Doppler of the CA performed in expiratory apnea that shows very high peak speeds (> 200 cm/s) due to severe stenosis

Color Doppler US can highlight the CA stenosis and Duplex Doppler US the consequent fluximetric variations such as the increase in the peak speed in the stenotic tract that can reach and exceed values of 200 cm/s (Fig. 16a–d) [Clip 6. DS. Duplex Doppler US which demonstrates the high-speed peaks (> 150 cm/s) due to stenosis of the CA]. MDCT can highlight the stenosis of the CA and the characteristic "Hooked appearance” that the CA assumes when it is compressed by the MAL (Fig. 17a, b). MRI can demonstrate both stenosis of the CA and the lower implant of the MAL (Fig. 18a, b).

### Treatment

Therapy consists of surgical treatment with open ligament release and celiac ganglionectomy [67, 68]. Surgery allows for rapid regression of symptoms in 85% of patients. In cases of recurrence (7%), treatment with endovascular stenting may be indicated [69].

## Conclusions

Ultrasound imaging plays an important role in the diagnosis of vascular compression syndromes. It allows you to significantly reduce false negatives and, in doubtful cases, provides indications for any further diagnostic analysis with second level methods. Failure to diagnose and treat, in these patients, could have serious consequences for their health.

## Data Availability

All data generated or analyzed during this study are included in this published article and its additional files.

## Abbreviations

TOS: Thoracic Outlet syndrome
US: Ultrasound
MRI: Magnetic Resonance Imaging
MDCT: Multidetector Computed Tomography
NCS: Nutckracker Syndrome
WS: Wilkie Syndrome
DS: Dunbar Syndrome
MALS: Mediane Arcuate Ligament Syndrome
CA: Celiac Artery
MAL: Mediane Arcuate Ligament
AO: Abdominal Aorta.
IVC: Inferior Cava Vein.
RRA: Right Renal Artery.
LRA: Left Renal Artery

## References

[1] Jones MR, Prabhakar A, Viswanath O (2019). Thoracic outlet syndrome: a comprehensive review of pathophysiology, diagnosis, and treatment. Pain Ther.

[2] Pesser N, Teijink JAW, Vervaart K et al. (2020) Value of Ultrasound in the Diagnosis of Neurogenic Thoracic Outlet Syndrome. Eur J Vasc Endovasc Surg. 10.1016/j.ejvs.2020.02.016 32199700

[3] Schut PC, Eggink AJ, Cohen-Overbeek TE (2020). Miscarriage is associated with cervical ribs in thoracic outlet syndrome patients. Early Hum Dev.

[4] Benzon HT, Rodes ME, Chekka K (2012). Scalene muscle injections for neurogenic thoracic outlet syndrome: case series. Pain Pract.

[5] Farina R, Foti PV, Iannace FA et al. (2019) Thoracic outlet syndrome: a rare case with bilateral cervical ribs and bilateral anterior scalene hypertrophy. J Ultrasound. 10.1007/s40477-019-00418-w PMC8363676 31834601

[6] Kaplan T, Comert A, Esmer AF (2018). The importance of costoclavicular space on possible compression of the subclavian artery in the thoracic outlet region: a radio-anatomical study. Interact Cardiovasc Thorac Surg.

[7] Sharma P, Rasheed I, Ansari MA (2010). Cervical rib causing thrombosis of subclavian artery. JNMA J Nepal Med Assoc.

[8] Fried SM, Nazarian LN (2013). Dynamic neuromusculoskeletal ultrasound documentation of brachial plexus/thoracic outlet compression during elevated arm stress testing. Hand (N Y).

[9] Bigler MR, Buffle E, Siontis GCM (2019). Invasive assessment of the human arterial palmar arch and forearm collateral function during transradial access. Circ Cardiovasc Interv.

[10] Fuhrman TM, Pippin WD, Talmage LA (1992). Evaluation of collateral circulation of the hand. J Clin Monit.

[11] Hixson KM, Horris HB, McLeod TCV (2017). The diagnostic accuracy of clinical diagnostic tests for thoracic outlet syndrome. J Sport Rehabil.

[12] Wilson MP, Low G, Katlariwala P (2020). Ultrasound for eurogenic thoracic outlet obstruction remains theoretical. Diagnostics (Basel).

[13] Wadhwani R, Chaubal JN, Sukthankar R (2001). Color Doppler and duplex sonography in 5 patients with thoracic outlet syndrome. Ultrasound Med..

[14] Chavhan GB, Batmanabane V, Muthusami P (2017). MRI of thoracic outlet syndrome in children. Pediatr Radiol.

[15] Ghouri MA, Gupta N, Bhat AP (2019). CT and MR imaging of the upper extremity vasculature: pearls, pitfalls, and challenges. Cardiovasc Diagn Ther.

[16] Svensson A, Brismar TB, Brehmer K (2020). Computed tomography venography of the upper extremities - Using low dose bilateral contrast media injection in a patient with suspected venous thoracic outlet syndrome. Radiol Case Rep.

[17] Chang KZ, Likes K, Davis K (2013). The significance of cervical ribs in thoracic outlet syndrome. J Vasc Surg.

[18] Rochlin DH, Orlando MS, Likes KC (2014). Bilateral first rib resection and scalenectomy is effective for treatment of thoracic outlet syndrome. J Vasc Surg.

[19] Vanti C, Natalini L, Romeo A (2007). Conservative treatment of thoracic outlet syndrome. A review of the literature. Eura Medicophys..

[20] Oh MJ (2017). Superior mesenteric artery syndrome combined with renal nutcracker syndrome in a young male: a case report. Korean J Gastroenterol.

[21] Wilkie DPD (1927). Chronic duodenal ileus. Am J Med Sci.

[22] De Macedo GL, Dos Santos MA, Sarris AB (2018). Diagnosis and treatment of the Nutcracker syndrome: a review of the last 10 years. J Vasc Bras.

[23] Gebhart T (2015). Superior mesenteric artery syndrome. Gastroenterol Nurs.

[24] Farina R, Pennisi F, Politi G (1999). Color Doppler-echo in Wilkie's syndrome. A case report. Radiol Med.

[25] Gulleroglu K, Gulleroglu B, Baskin E (2014). Nutcracker syndrome. World. J Nephrol.

[26] Genov PP, Kirilov IV, Hristova IA (2019). Management and diagnosis of Nutcracker syndrome-a case report. Urol Case Rep.

[27] Mauceri B, Misseri M, Tsami A (2010). Ultrasound in diagnosis of superior mesenteric artery syndrome. Clin Ter.

[28] Cicero G, D’Angelo T, Bottari A (2018). Superior mesenteric artery syndrome in patients with crohn’s disease: a description of 2 cases studied with a novel magnetic resonance enterography (MRE) procedure. Am J Case Rep.

[29] Warncke ES, Gursahaney DL, Mascolo M (2019). Superior mesenteric artery syndrome: a radiographic review. Abdom Radiol (NY).

[30] Di Matteo F, Picconi F, Sansoni I (2010). Superior mesenteric artery syndrome diagnosed with linear endoscopic ultrasound. Endoscopy..

[31] Agrawal GA, Johnson PT, Fisherman EK (2007). Multidetector row CT of superior mesenteric artery syndrome. J Cin Gastroenterol.

[32] Er A, Uzunlulu N, Guzelbey T, Yavuz S (2019). The nutcracker syndrome: The usefulness of different MRI sequences for diagnosis and follow-up. Clin Imaging.

[33] Wong HI, Chen MC, Wu CS (2010). The usefulness of fast-spin-echo T2-weighted MR imaging in Nutcracker syndrome: a case report. Korean J Radiol.

[34] Farina R, Foti PV, Cocuzza G (2017). Wilkie's syndrome.. J Ultrasound.

[35] Shin JI, Baek SY, Lee JS (2007). Follow-up and treatment of nutcracker syndrome. Ann Vasc Surg.

[36] Jain N, Chopde A, Soni B et al. (2020) SMA syndrome: management perspective with laparoscopic duodenojejunostomy and long-term results. Surg Endosc. 10.1007/s00464-020-07598-1 32342220

[37] Agle CG, Amorim DS, De Almeida LC (2019). Endovascular treatment of Nutcracker syndrome: case report. J Vasc Bras.

[38] Wang He, Guo Y-T, Jiao Y (2019). A minimally invasive alternative for the treatment of nutcracker syndrome using individualized three-dimensional printed extravascular titanium stents. Chin Med J (Engl).

[39] May R, Thurner J (1957). The cause of the predominantly sinistral occurrence of thrombosis of the pelvic veins. Angiology.

[40] Du Pont B, Verbist J, Van den Eynde W (2016). Right-sided Cockett's syndrome. Acta Chir Belg.

[41] Cheng L, Zhao H, Zhang FX (2017). Iliac vein compression syndrome in an asymptomatic patient population: a prospective study. Chin Med J (Engl).

[42] Molloy S, Jacob S, Buckenham T (2002). Arterial compression of the right common iliac vein; an unusual anatomical variant. Cardiovasc Surg.

[43] Farina R, Foti PV, Iannace FA et al. (2020) May Thurner syndrome: description of a case with unusual clinical onset. J Ultrasound. 10.1007/s40477-020-00497-0 PMC9148327 32577934

[44] Palma L, Peterson MD, Ingebretsen R (1995). Iliac vein compression syndrome from urinary bladder distension due to prostatism. South Med J.

[45] Rosengarten AM, Wong J, Gibbons S (2002). Endometriosis causing cyclic compression of the right external iliac vein with cyclic edema of the right leg and thigh. J Obstet Gynaecol Can.

[46] Justa DG, Bianco FJ, Ogle A (2003). Deep venous thrombosis due to compression of external iliac vein by the penile prosthesis reservoir. Urology.

[47] Janczak D, Rucinski A, Skora J (2000). Iliac-femoral vein thrombosis as a first symptom of the isolated common and internal illiac artery aneurysm. Wiad Lek.

[48] Rosenthal D, Matsuura JH, Jerius H (1998). Iliofemoral venous thrombosis caused by compression of an internal iliac artery aneurysm: a minimally invasive treatment. J Endovasc Surg.

[49] Liyanage AM, Shafiq T, Wadekar VR (2018). An Unusual Presentation of Deep Vein Thrombosis. Eur J Case Rep Intern Med.

[50] Qian AM, Cai ZX, Zhang S (2019). Endovascular treatment for non-thrombotic right iliac vein compression syndrome with intravascular ultrasound. Zhonghua Yi Xue Za Zhi.

[51] Bondarev S, Keller EJ, Han T (2019). Predictors of Disease Recurrence after Venoplasty and Stent Placement for May-Thurner Syndrome. J Vasc Interv Radiol.

[52] Gavrilov SG, Vasilyev AV, Krasavin GV et al. (2020) Endovascular interventions in the treatment of pelvic congestion syndrome caused by May-Thurner syndrome. J Vasc Surg Venous Lymphat Disord. 10.1016/j.jvsv.2020.02.012 32241734

[53] Barge TF, Wilton E, Wigham A (2020). Endovascular treatment of an extensive iliocaval and renal vein thrombosis secondary to inferior vena cava stenosis and May-Thurner type iliac vein compression: a case report. Vasc Endovascular Surg.

[54] Lopez R, DeMartino R, Fleming M (2019). Aspiration thrombectomy for acute iliofemoral or central deep venous thrombosis. J Vasc Surg Venous Lymphat Disord.

[55] Xu F, Tian Z, Huang X (2019). A case report of May-Thurner syndrome induced by anterior lumbar disc herniation: Novel treatment with radiofrequency thermocoagulation. Medicine (Baltimore).

[56] Santos GM, Viarengo LMA, Oliveira MDP (2019). Celiac artery compression: Dunbar syndrome. J Vasc Bras.

[57] Camacho N, Alves G, Bastos Gonçalves F (2017). Median arcuate ligament syndrome - literature review and case report. Rev Port Cir Cardiotorac Vasc.

[58] Bech F, Loesberg A, Rosenblum J (1994). Median arcuate ligament compression syndrome in monozygotic twins. J Vasc Surg.

[59] Ali M, Patel J (2016). Dunbar syndrome following liver transplantation. BMJ Case Rep..

[60] Köhler M, Schardey HM, Bettels R (2018). Median arcuate ligament syndrome - imaging presentation and interdisciplinary management. Rofo.

[61] Sunkara T, Caughey M, Cai ZK (2017). Dunbar syndrome- a rare cause of foregut ischemia. J Clin Diagn Res..

[62] Saleem T, Baril DT (2020). Celiac artery compression syndrome. StatPearls.

[63] Acampora C, Di Serafino M, Iacobellis F et al. (2020) Insight into Dunbar syndrome: color-Doppler ultrasound findings and literature review. J Ultrasound. 2020. 10.1007/s40477-019-00422-0 PMC8363698 31925730

[64] Patel MV, Dalag L, Weiner A (2019). Inability of conventional imaging findings to predict response to laparoscopic release of the median arcuate ligament in patients with celiac artery compression. J Vasc Surg.

[65] Klimas A, Lemmer A, Bergert H (2015). Laparoscopic treatment of celiac artery compression syndrome in children and adolescents. Vasa.

[66] Berek P, Kopolovets I, Dzsinich,  (2018). Celiac axis compression syndrome - diagnostic and surgical treatment. Rozhl Chir Summer.

[67] Grus T, Klika T, Grusová G (2018). Dunbar syndrome - single-center experience with surgical treatment. Rozhl Chir Winter.

[68] Torres OJM, Gama-Filho OP, Torres CCS (2017). Laparoscopic treatment of Dunbar syndrome: a case report. Int J Surg Case Rep.

[69] Hongsakul K, Rookkapan S, Sungsiri J (2012). A severe case of median arcuate ligament syndrome with successful angioplasty and stenting. Case Rep Vasc Med.

